# Epigenetic: a molecular link between testicular cancer and environmental exposures

**DOI:** 10.3389/fendo.2012.00150

**Published:** 2012-11-29

**Authors:** Aurelie Vega, Marine Baptissart, Françoise Caira, Florence Brugnon, Jean-Marc A. Lobaccaro, David H. Volle

**Affiliations:** ^1^Génétique Reproduction et Développement, INSERM U 1103Aubière, France; ^2^Génétique Reproduction et Développement, Clermont Université, Université Blaise PascalClermont-Ferrand, France; ^3^Génétique Reproduction et Développement, CNRS, UMR 6293Aubière, France; ^4^Centre de Recherche en Nutrition Humaine d’AuvergneClermont-Ferrand, France

**Keywords:** testicular cancer, physiology, epigenetic, endocrine disrupters

## Abstract

In the last decades, studies in rodents have highlighted links between *in utero* and/or neonatal exposures to molecules that alter endocrine functions and the development of genital tract abnormalities, such as cryptorchidism, hypospadias, and impaired spermatogenesis. Most of these molecules, called endocrine disrupters exert estrogenic and/or antiandrogenic activities. These data led to the hypothesis of the testicular dysgenesis syndrome which postulates that these disorders are one clinical entity and are linked by epidemiological and pathophysiological relations. Furthermore, infertility has been stated as a risk factor for testicular cancer (TC). The incidence of TC has been increasing over the past decade. Most of testicular germ cell cancers develop through a pre-invasive carcinoma *in situ* from fetal germ cells (primordial germ cell or gonocyte). During their development, fetal germ cells undergo epigenetic modifications. Interestingly, several lines of evidence have shown that gene regulation through epigenetic mechanisms (DNA and histone modifications) plays an important role in normal development as well as in various diseases, including TC. Here we will review chromatin modifications which can affect testicular physiology leading to the development of TC; and highlight potential molecular pathways involved in these alterations in the context of environmental exposures.

## TESTICULAR CANCERS

Testicular cancer (TC) is a rare solid tumor which account for 1% of cancers in men. However, it is the most common cancer in men in their 20s and 30s (Ziglioli et al., 2011). It is a real sanitary problem as it affects men during their reproductive years. Interestingly, the development of TC has been associated with urogenital abnormalities (Olesen et al., 2007). TC development has been associated with cryptorchidism, hypospadia, and hypo-fertility. Indeed, epidemiological studies argue for an increased risk of testicular germ cell tumor in males who suffer for fertility troubles (Burns et al., 2010).

### EPIDEMIOLOGY

In developed countries, the prognosis for TC is excellent, with a 5-year survival rate >95%. Despite it represents the most frequent solid cancer in young men, it seems a quite rare pathology (Richardson et al., 2012). Thus all these data has led to consider testicular tumors as a curable neoplasm. It has to be noted that there are some geographic and racial variation in the risk of TC development (Richiardi et al., 2004). Indeed, in US, Hispanic men had the largest increase in incidence rates for non-seminomas (Townsend et al., 2010). Next to this, the genetic predisposition has been suggested as men with familial history of germ cell TC (TGCC) have a 4- to 10-fold increased risk to develop also such tumor (Greene et al., 2010). The analysis of this genetic risk seems to be associated with an autosomal recessive mode of inheritance that must result of the combined effects of several alleles. It was estimated that 25% of TC susceptibility account for by genetic effects (Turnbull and Rahman, 2011). If TCs are quite rare in African men, the morbidity and mortality of TC is quite high in developing countries (Ugwumba and Aghaji, 2010). These data highlight the impact of poverty and paucity of resources to detect and treat cancers. In that line, it has been reported that socioeconomic position has an impact on the risk of TC development as well as the survival rate (Richardson et al., 2012).

The main way to identify TC as soon as possible is auto-palpation by men to look at increased size or the appearance of “hard” testis (Umeh and Chadwick, 2010). After identification of the anomalies, biopsies will help to characterize the type of TC. Then imaging will also play a key role to determine the lymphatic extension and potential metastasis (Brunereau et al., 2012).

There are several kinds of TCs that need to be characterized by the physician using anatomopathological approach. There are several kinds depending on cell type at the origin of the development of TC (van de Geijn et al., 2009); thus there are intratubular germ cell neoplasia (IGCN), atypical germ cells with maturation arrest (MA), pseudolymphovascular invasion, real lymphovascular invasion in germ cell tumors, macroscopic Sertoli cell nodules, and Sertoli cell tumors. However, we can thus distinct germ cell tumors versus non-germ cell tumors. The most common TC are germ cell one which represent 95% of these tumors. There are two main types described which are seminoma and non-seminomatous germ cell tumors, with a third one which is the spermatocytic seminoma (Mannuel et al., 2012).

Here we will focus this review on germ cell cancers. Their incidence has been reported to be continuously increasing in the last decades in men of different industrialized countries.

Regarding the two major groups of testicular germ cell cancers which are the seminomas and non-seminomas, each represent approximately 50% of germ cell tumors. It is interesting to note that around 15% of TGCC contain a mix of seminoma and non-seminomatous tumors (Aschim et al., 2006).

Seminomas are managed with orchiectomy and surveillance or radiotherapy in stage I and chemotherapy in more advanced stages (Warde et al., 2011). On the other side, non-seminomas which consist of several histologic types, such as choriocarcinoma, teratoma, yolk sac tumor, and embryonal cell carcinoma, each with different tumor marker profile, are less sensitive to radiation and are frequently treated with chemotherapy (Warde et al., 2011).

### GENETIC ASSOCIATION

The development of TGCC is associated with many chromosomal abnormalities (Summersgill et al., 1998). Indeed, TGCC are aneuploid; with non-seminomatous tumors and seminoma being respectively hypotriploid and hypertriploid. The major association is with the gain of material from the chromosome arm 12p in both seminoma and non-seminomatous tumors (Atkin and Baker, 1982). The analyses of these chromosomal reorganizations lead to the characterization of potential candidates involved in the TGCC pathology. Among them, there is amplification of the KRAS and Cyclin d2 genes (Rodriguez et al., 2003). They are associated with malignant transformation and proliferation. Next to these genes, there are also those implicated in the cell pluripotency such as Stella and Nanog (Clark et al., 2004).

The strongest association for TGCC susceptibility is for single nucleotide polymorphisms (SNPs) at the 12q22 within the *kit-ligand* gene (Kanetsky et al., 2009). It is correlated with a 2.5-fold increased risk of disease. This gene has been involved in several aspect of primordial germ cell (PGC) development. Indeed, it seems to act on PGC migration and survival (Gu et al., 2009). These impacts might rely on the downstream target KRAS which then activate the p110 catalytic subunit of the PI3K pathway which in turn through AKT pathways will act on proliferation, survival, and migration (Sasaki et al., 2003). KRAS could also mobilize the MAPK pathways reinforcing its impact on proliferation, survival, and migration processes.

Next to this, as testicular physiology is under the control of the endocrine functions mainly through the activity of androgen and estrogen receptors, it was deeply studied if polymorphisms of genes involved in hormonal metabolism could be associated with a higher risk of TCs. Even if some reports are contradictory, the studies focused on the androgen receptor (AR), the estrogen receptors and genes involved in either synthesis or degradation of the hormones.

Regarding the estrogen receptors, it was demonstrated that polymorphisms in ERα are associated with azoospermia (Romerius et al., 2011) and are more likely to be associated with the risk of seminoma and metastasis (Brokken et al., 2012); whereas polymorphisms in ERβ are more likely to be link to altered spermatogenesis (Aschim et al., 2005) and with risk of TGCC. New to this, polymorphisms in 17-β hydroxydehydrogenase-4 which convert androgen and estrogen to weaker hormones were associated with TGCC (Chia et al., 2010; Ferlin et al., 2010). In these metabolic pathways, polymorphisms in cytochrome P450 Cyp-1A1 gene, encoding a hormone-metabolizing protein, were identified and inversely correlated with TC. Their effects were more or less severe regarding the different polymorphisms suggesting that it may contribute to susceptibility to TGCC development (Figueroa et al., 2008; Kristiansen et al., 2011).

In this hormonal context, one of the most studied genes in regards to polymorphisms is the AR. The AR gene has two polymorphic regions in exon-1 with CAG codon encoding for glutamine and GCN which encode for glycine. Changes in the length of these polymorphic trinucleotide repeats, (CAG) and/or (GGN), lead to altered transactivation of the AR which has been shown to play a role in several forms of endocrine cancer such as prostate cancer. Regarding TC some studies are a bit contradictory showing either or not link with increased risk of TGCC (Rajpert-De Meyts et al., 2002; Garolla et al., 2005). However, it appears that the increased risk of seminoma was associated with the shorter CAG repeat length. This suggests that an increased AR transactivation may be involved in the development of seminoma and/or progression of carcinoma *in situ* (CIS) to seminoma (Davis-Dao et al., 2011). It is also demonstrated that the combination of altered number in repeat for both CAG and CGC is important for the correlation with TC. Indeed, Garolla et al. (2005) showed that the combination of CAG (20 repeats) and GGC (17 repeats) was more frequent in patient with TGCC (8 versus 1.7% in control patients).

Like most of the cancer pathologies, TCs seem to be the results of either genetic and environmental factors. It has been stated that TCs derive from a precocious lesion, the CIS of the testis, also known as IGCN or testicular intraepithelial neoplasia (TIN; Sonne et al., 2008). This lesion deserves great attention, because the diagnosis of CIS may lead to a precocious diagnosis of TCs. Usually, the diagnosis of CIS is incidental.

If there is a consensus on the fact that the process of the TC pathology may found its origin during embryonic life of the individual, it can also be suspected that there might also be some other events participating to its appearance. Indeed, it seems quite a long process that TC occurs in the 20s or 30s of life when first event took place in fetal life. Thus it could be hypothesized that there might be a second hit at puberty, like hormone burst that could provoke the definitive occurrence of TC. This clearly highlights the importance of the microenvironment. A study on patients with testicular maldescent shows that there is around a two-fold increased risk to develop TGCC if the orchidopexy is performed after the age of 13, compare to men who had the operation before their 13 (Walsh et al., 2007). Thus placing the testes in the scrotum before puberty decreases the incidence of TGCC; suggesting that intra-abdominal location at puberty promotes testicular tumorigenesis. Gene expression profiling carried out on TGCC samples demonstrate marked differences between the histological subtypes of TGCC. This reflects stages and patterns of differentiation. It also supports a model of differentiation from IGCN to seminomas or embryonal carcinomas. Gene expression patterns and genomic imprinting analyses in TGCC show similarity to PGCs and gonocytes (Ziglioli et al., 2011).

## ENVIRONMENTAL ISSUES

### THE TESTICULAR DYSGENESIS SYNDROME HYPOTHESIS

In 1993, Skakkebaek and colleagues proposed that various disorders of male reproductive health, namely cryptorchidism, hypospadias, subfertility, and testicular germ cell tumor, derived from perturbations of embryonal programing and gonadal development during fetal life (Sharpe and Skakkebaek, 1993). Thus they defined it as testicular dysgenesis syndrome (TDS; Skakkebaek et al., 2001). The anomalies may lead to early symptoms, such as hypospadias and undescended testis, as well as late effects such as testis cancer and infertility. The most frequent abnormality due to TDS may be impaired spermatogenesis.

A fetal origin is obvious with regard to two symptoms of TDS: hypospadias and cryptorchidism. Moreover, studies suggested that the precursor cells of testis cancer, CIS testis, are similar to fetal gonocytes. The accepted etiology for germ cell cancer suggests that developmental arrest of fetal germ cell differentiation is a main event leading to persistence of gonocytes, which in turn develop into CIS. The causes remain unknown, although disturbances in the microenvironment provided by the Sertoli and Leydig cells may play a major role. Moreover, spermatogenesis is strictly controlled and depends on a succession of signals provided by the local environment (Skinner et al., 1991; Verhoeven, 1992; Jégou, 1993).

Among all the studies on testicular functions, there are several windows of time that must be critical for its development. Next to the importance of the fetal development, it appears that puberty must be an important timing. This is when hormone levels will reach optimal concentrations leading to the secondary sexual characters. This will also coincide with the appearance of the TGCC, as men are affected between 15 and 35 years old.

Hormones play major impacts on testicular functions throughout the life of the individuals. Indeed, testis is a key target for androgen and estrogen actions. These hormonal sensitivities have been studied for decades (Verhoeven et al., 2010). The role of testosterone is evident in patients with complete androgen insensitivity syndrome (Sultan et al., 1993).

Moreover, testicular descent occurs in two phases: the transabdominal phase, which animal studies suggest depends on the insulin-like hormone 3 (INSL3) produced by interstitial Leydig cells (Kaleva and Toppari, 2005; Ferlin et al., 2006, 2009). It has been demonstrated that the expression of the Insl3 gene is under the control of the estrogen signaling pathway (Cederroth et al., 2007). Then, the inguinoscrotal phase is dependent on androgens.

Androgens play a crucial role in the control of spermatogenesis. Molecular details have been discovered using transgenic mice invalidated (knock-out, KO) for AR either in the testis or in different testicular compartments. Such mice have low testosterone levels with altered expression of steroidogenic enzymes, even Leydig cell mass is altered (Wang et al., 2009). AR is involved in autocrine action of testosterone on Leydig cells. Testosterone deficiency is responsible for spermatogenesis arrest due to altered Sertoli functions (Wang et al., 2009).

In mouse ERα have been demonstrated to be involved in the maturation of the spermatozoa (Lubahn et al., 1993). These KO mice present an excess of fluid which increases the pressure within the seminiferous tubules and leads to the germ cell loss (Eddy et al., 1996; Hess et al., 1997). Surprisingly, the ERβ-KO mice show no testicular phenotype (Couse and Korach, 1999).

The deficient male mice for Cyp19 (Cyp19-KO), encoding for the enzyme responsible for the aromatization of testosterone into estrogens, develop normally and their genital tract is anatomically similar to that of the wild-type (Robertson et al., 1999). Males are fertile; however, some of Cyp19-KO males exhibit an altered spermatogenesis by the age of 5 months (Robertson et al., 1999). By the age of 1 year, all males develop abnormal spermatogenesis with a blockage of germ cell maturation at the spermatid stage. This is due to an increase in apoptotic rates when compared to the wild-type animals. The observation of abnormal acrosome development in the Cyp19-KO mouse suggests that acrosome biogenesis could be estrogen-dependent (Robertson et al., 1999). Interestingly, estradiol have been demonstrated to play a role as a survival factor for the human germ cells (Pentikäinen et al., 2000), and also is beneficial for sperm motility (Carreau and Hess, 2010). Moreover, next to these data, deleterious effects of numerous endocrine disruptors on sperm count and male genital tract (cryptorchidism, hypospadia, and infertility) have been documented (Iguchi et al., 2001; Sikka and Wang, 2008) particularly in the context of *in utero* and/or neonatal exposures.

Imbalanced equilibrium between the estrogen and androgen levels *in utero* is hypothesized to influence TC risk. Thus, alterations in genes involved in the action of sex hormones may contribute to variability of an individual’s susceptibility to TC. Mutations in testosterone pathway genes may alter the level of testosterone *in vivo* and hypothetically the risk of developing TC (Kristiansen et al., 2012). In regard with the hypothesis of the TDS and the known impact of steroids on testicular development and functions, it has been hypothesized that endocrine-disrupting chemicals could play a role in these pathologies.

### ENDOCRINE DISRUPTERS

The endocrine disrupters (EDs) are compounds which may be of industrial or natural origin and which act to dysregulate steroid function and metabolism. They produce their effects by mimicking, antagonizing, or altering endogenous steroid levels (androgens or estradiol, E2) *via* changing rates of their synthesis or metabolism and/or expression or action at receptor targets.

The question of environmental endocrine disruption has been a topic of public concern for many years and remains high on the scientific agenda. Indeed the number of chemical used is constantly increasing in developed countries, it is supposed that humans and animals can be exposed to a growing number of contaminants which can accumulate in their bodies and may have adverse consequences for health. Large range of chemicals (banned or still in use) have been characterized as EDs. Among these EDs, highly produced, Bisphenol A (BPA; Rubin, 2011) is present in plastics, including beverage and food storage containers and in the ink used for thermal paper receipts. Some individuals have also been exposed to contaminants with adverse effects originating from medical use (diethylstilbestrol, DES; Bullock et al., 1988), or dietary habits (phytoestrogens; Naciff and Daston, 2004). More surprisingly, in China, human excretions were suspected to be the major contributor of estrogens in municipal wastewater (Zhou et al., 2012).

Moreover, studies have made correlations between elevated levels of phthalates in urines of pregnant women and lower masculinization of their progeny (Suzuki et al., 2012). EDs have also been detected in the maternal milk (Hines et al., 2009).

Regarding the urogenital development and more particularly testis, the impacts of EDs have been quite well described on human and rodent models (Volle et al., 2009; Toppari et al., 2010; Desdoits-Lethimonier et al., 2012). This is mainly true for hypospadia, cryptorchidism, and infertility; but the link with TGCC remain to be defined.

It has been demonstrated that EDs with estrogenic activity lead to a decrease of expression of steroidogenic genes which by the end result in lower testosterone concentrations (Joensen et al., 2008). However, the mechanisms seem to differ between species. This highlights the difficulties to find good experimental models. Indeed, one will claim that human cells will not react as murine cells; and other may assume that cell lines or explants tissues might not totally react as in the body. Indeed, endocrine systems are really complex and need to be analyzed in regard of the complexity of integrative physiology.

The effect of such EDs in human pathology is quite difficult to establish. It is really difficult to have standardized cohort between the studies, which is due to the history of patients (environmental and genetic factors). This heterogeneity leads to inconsistent studies which do not allow definitive conclusions on the potential involvement of EDs in TGCC.

It has been demonstrated that there was a six-fold increase in the risk of seminoma among plastic workers exposed to polyvinyl chloride (PVC; Ohlson and Hardell, 2000). However, Hardell et al. (2004a) did not found association between PVC exposure and TC.

Another study from Hardell et al. (2006) show that chlorinated biphenyls (PCBs) could play a role during fetal exposure in the etiology of TC as case mothers were identified to have significantly higher concentrations of these PCBs. However, regarding PCBs, no different risk pattern could be demonstrated for seminoma and non-seminomatous TC (Hardell et al., 2003, 2004b).

However, several studies have demonstrated some effects of these molecules on risk of TGCC (Hardell et al., 2003, 2004b, 2006; McGlynn et al., 2009). In their study McGlynn et al. (2009) associated PCBs with a decrease risk of TGCC development. Different PCBs are either associated with decrease risk of either seminoma or non-seminoma. In the same line of evidence, it was demonstrated that in patients with seminoma there was differences in concentrations of different PCBs (Purdue et al., 2009). Some PCBs (44, 49, and 52) were found to be lower accumulated in patients with seminoma compare to congeners; whereas other PCBs (99, 138, 153, 167, 183, and 195) were significantly higher accumulated in these patients. This suggest that rather than specific concentration of one or another PCB, it may be the relative concentrations of multiple one that might be relevant to established clear correlation with the risk of TC.

Moreover, evidences suggest that exposures to pesticides could be risk factor of TC development. Indeed, a recent study reported a significant association between TC development and use of insecticides such as organochlorine pesticides, namely dichlorodiphenyldichloroethylene (p,p′-DDE) isomer and hexachlorobenzene (Giannandrea et al., 2011).

Next to this, as TC development seems to be either associated with genetic predisposition and or environmental exposure, it might be of interest to analyze the potential combination of such anomalies. Consistent with this hypothesis, as polymorphisms in AR and some organochlorine pesticides have been associated to risk of TGCC development, and that some of these organochlorine pesticides present anti-androgenic activities, Biggs et al. (2008) have studied the potential interaction of AR polymorphisms and exposure to p,p′-DDE and the association with TC risk. According to their study, they were not able to demonstrate any association between p,p′-DDE exposure and TC risk, either or not in combination with (CAG)n length. However, a point to take in consideration is that p,p′-DDE concentration was measured in adult patients, but as TC is supposed to found its origin in fetal life, this must be the exposition in this period of life that must be significant to define such association. However, it will be difficult for instance to establish such correlation as we do not have access to fetal/neonatal blood of these patients. However, it could be though to perform some clinical trials to collect blood of new borns and make assay for pesticides concentrations. Then it will be necessary to follow these boys and see if they will further develop TC, and perform correlation with fetal/neonatal concentrations of pesticides.

#### ED and insulin-like-3

Next to their steroidogenic function, Leydig cells during development express the insulin-like-3 gene which is responsible for gubernaculum maturation (Ivell and Anand-Ivell, 2011). In human, INSL3 is produced by prenatal, neonatal, and adult Leydig cells to various extents (Ivell and Anand-Ivell, 2011). INSL3 production seems to be dependent on the state of Leydig cell differentiation, and is stimulated by the long-term trophic effects mediated by luteinizing hormone (LH; Toppari et al., 2007). This finding clearly helps in understanding the complete process of testis descent. The role of the INSL3 on testis descent was highlighted by the fact that mouse KO for the gene encoding *Insl*3 results in cryptorchidism (Adham and Agoulnik, 2004). Moreover, animal model of ED exposures demonstrated that INSL3 production is sensitive to estrogenic or anti-androgenic compounds. This clearly suggests that maternal exposure to EDs during pregnancy can result in cryptorchidism, a factor that predispose to TC development.

#### ED and steroidogenesis

This link is of particular importance. Indeed, if CIS have been described in boys at birth (Jacobsen and Henriques, 1992), TCs appear in young men between 15 and 35 years old, suggesting that puberty and probably the increase of hormone concentrations must be key events.

The impact of EDs on steroidogenesis has been demonstrated for several decades now. EDs have been described to inhibit critical cellular functions involved in steroidogenesis, such as transport of cholesterol into the mitochondria, the expression of steroidogenic genes or the activity of these enzymes (Vanparys et al., 2012). Indeed, it has been demonstrated that exposure of adult rats to 2,3,7,8-tetrachlorodibenzo-*p*-dioxin (TCCD) led to lower production of testosterone in response to human choriogonadotropin (hCG), relative to testes from control rats (Kleeman et al., 1990). The addition of pregnenolone is able to restore normal testosterone secretion. This highlights that the impact on steroid synthesis might results from altered cholesterol synthesis or mobilization to the mitochondria (Moore et al., 1991). TCDD was also observed to reduce the number and size of the Leydig cells (Johnson et al., 1994). Moreover, the use of primary culture of Leydig cells also demonstrate that TCDD represses *Cyp11a1* expression, through the alteration of the ability of hCG to increase intracellular cAMP levels (Lai et al., 2005).

The impact on the animal phenotypes will depend on the age at exposure. Thus fetal/neonatal exposure will lead to altered internal genital organs. Indeed, experimental models have shown that disruption of the androgen signaling results in feminization of external genitalia (Nagahama et al., 2004). In animal models, exposures to low concentrations of EDs are able to alter testosterone synthesis without any effect of feminization. However, the most interesting thing is that even after neonatal exposure, the adult testosterone concentrations are decreased suggesting long-term impact that is specific to this particular window of exposure (Volle et al., 2009). This has been clearly highlighted as the exposition of adult animal lead to transitory testosterone decrease, associated with temporary germ cell death. In human, majority of the results relies on epidemiological studies and correlative data (Woodruff, 2011). However, several studies using testicular explant models have tried to characterize the impact of EDs on human Leydig cells (Desdoits-Lethimonier et al., 2012). By the end, all these data led to the idea that fetal cells must be more sensitive than adult Leydig cells.

Regarding the signaling pathways involved, and as main regulators of the expression of steroidogenic genes, a reduced activity of the cAMP-mediated PKA pathway would also be expected to reduce the mobilization of cholesterol by cholesterol hydrolases. However, at the molecular level, it has been demonstrated in mouse that neonatal exposure to EDs with estrogenic activity leads to a decrease of testosterone synthesis through ER receptors and that the orphan nuclear receptor SHP must be a key intermediary for this effect (Volle et al., 2009). Indeed, the SHP KO mice seem to be less sensitive to estrogenic EDs than their wild-type littermates. It was also demonstrated that the impact of SHP on steroidogenesis is due to the repression of either expression and/or activity of the nuclear receptors Sf-1 and Lrh-1 (Volle et al., 2009). It is to note that SHP was not involved in early post-natal decrease of testosterone production induced by estrogenic EDs. This highlights that the involved molecular pathways affected by EDs are multiple and complex.

#### ED and germ cell differentiation

Spermatogenesis, leading to spermatozoa formation, is a complex process with multiple steps involving mitosis, meiosis, and spermiogenesis. It takes place in the seminiferous tubules. There, germ cells are organized from the base of the tubule to the lumen. Germ cells are supported by the nursing Sertoli cells, which extend from the base to the lumen of the seminiferous tubules. Efficient spermatogenesis also relies on the integrity of tight junctions between the Sertoli cells which form the blood–testis barrier (BTB; Cheng and Mruk, 2012). BTB has many functions in the testis such as maintaining a particular immune context and also the control of the flow of nutrients and growth factors that are required for the development of germ cells.

The key role of retinoids in the differentiation process of germ cells was highlighted by two studies demonstrating stra8 as a key factor (Bowles et al., 2006; Koubova et al., 2006). It also involved the timely regulation of the gene encoding for cyp26b1, an enzyme responsible for retinoid degradation. Indeed, CYP26B1 in Sertoli cells acts as a masculinizing factor to arrest male germ cells in the G0 phase of the cell cycle and prevents them from entering meiosis (Li et al., 2009). This induction of retinoid pathway to induced entry in meiosis seems to be inhibited by the nuclear receptor SHP, a co-repressor of the retinoid acid receptors RAR (Volle et al., 2007). In the opposite manner, the FGF9 signaling pathway acts to determine germ cell fate to enter meiosis (Boisen et al., 2001).

## EPIGENETIC AND TESTICULAR CANCERS

### TRANSGENERATIONAL EFFECTS: ROLE OF EPIGENETIC MODIFICATIONS

Epigenetic refers to changes of DNA information without any change of the sequences. It relies on histones post-transductional modification (acetylation, methylation...) or of DNA methylation levels. Thus, inheritance of these information requires germline transmission of epigenetic patterns between generations. The epigenetic programing of the germline occurs during embryonic development in a sex-specific manner (Western, 2009). These processes are crucial for reproductive functions, as most of mouse models with specific germline invalidation for gene responsible of DNA or histones modifications led to sterile animals (Peters et al., 2001; La Salle et al., 2007). Regarding the male germline, it becomes imprinted following sex determination. Thus after puberty when spermatogenesis is fully functional, there are also specific epigenetic modifications along the different steps of germ cell differentiation from spermatogonial precursors up to testicular spermatozoa. Indeed, it has been demonstrated that throughout the different steps of spermatogenesis, germ cells have a dynamic of the epigenetic modifications. This is highlighted by the changes in the expression levels of the enzymes involved in these modifications. If DNA methyltransferases (Dnmts) are mainly expressed in the spermatogonia, histone methyltransferases (HMTs) are mainly expressed at the spermatocyte levels (Godmann et al., 2009). Then other histone modifications such as hyperacetylation of the H4 histone play a key role in the removal of histones and their replacement by protamines during spermatogenesis (Dhar et al., 2012). Such epigenetic modifications have also been demonstrated to be associated with infertility as for example reduced expression of *Dnmt3b* in patients with spermatogenic arrest (Adiga et al., 2011).

If the EDs cannot alter the DNA sequence, there are numerous of studies demonstrating that they can impact the epigenome. Indeed, environmental factors can alter the epigenetic programing which will impact the development of the offsprings. Interestingly, it has been demonstrated in the last decade that these effects could also potentially impact the subsequent generations (Huang et al., 2011; Schoevers et al., 2012). Such effect was first demonstrated for the exposure with vinclozolin, a compound with anti-androgenic activities (Anway et al., 2005). Thus exposure during embryonic gonadal sex determination can alter the male germline epigenetics (e.g., DNA methylation). The epigenetic mechanism involves the alteration of DNA methylation in the germline that appears to transmit transgenerational adult onset disease, including spermatogenic defects and cancer (Anway et al., 2005). At the molecular level, the authors identify by global transcriptomic approach that the expression of genes involved in these processes such as the Dnmt or responsible for histone modifications was affected by vinclozolin treatment (Anway et al., 2005). All these data suggest the main involvement in transgenerational transmission.

#### Potential link between epigenetic and TGCT?

These modifications in epigenetic programing by EDs are of particular interest as they have been suspected to be responsible of the increased rate of pathologies related to the TDS such as TCs. This potential association between ED and epigenetic in TGCT development is highlighted by the fact that recently, number of studies have demonstrated the modified epigenetic profile of tumor cells compare to “normal” cells (Okamoto and Kawakami, 2007; Brait et al., 2012). Indeed, it appears that tumor cells show an epigenetic pattern similar to undifferentiated spermatogonial stem cells (Almstrup et al., 2010). Indeed, CIS cells have a permissive and fetal-like chromatin structure (Almstrup et al., 2010). This is consistent with the fact that CIS, precursor of TGCT have already been identify in testis of embryos.

***DNA methylation***. DNA methylation is an important event during germ cell development. These enzymatic modifications of DNA rely on Dnmts. Among them, expression profiles of DNMT3a and DNMT3l suggest that they might act during prenatal germ cell development for the establishment of *de novo* methylation. On the other side, DNMT1 and DNMT3b rise shortly after birth in the male (Godmann et al., 2009). It was thus hypothesized that these two Dnmts must be involved in the maintenance of methylation patterns in proliferating spermatogonia.

The use of mouse models invalidated for genes encoding these enzymes highlights their crucial involvement during spermatogenesis. Dnmt3a^-^^/^^-^ males show a greatly reduced number of spermatocytes (Yaman and Grandjean, 2006; La Salle et al., 2007). This suggests a major involvement for progression through meiosis. In the same line, the males invalidated for Dnmt3L participate to the acquisition of DNA methylation at paternally imprinted regions, unique non-pericentric heterochromatic sequences, and interspersed repeats, including transposable elements. Moreover, Dnmt3L^-^^/^^-^ males present alterations of meiotic process leading to spermatogenesis arrest, and spermatocytes apoptosis (Webster et al., 2005). As Dnmt3L expression is restricted to gonocytes, the presence of defects in later stages suggests alteration of processes required for completion of spermatogenesis.

The major role of these epigenetic alterations has been demonstrated in carcinogenesis. Indeed, it has been shown that DNA methylation is associated with repression of tumor suppressor gene expression. This epigenetic process is one of the most studied in the research field, and has been recognized as a major mechanism during TGCC progression (Manton et al., 2005; Ellinger et al., 2009). Moreover, the DNA methylation pattern seems to correlate with histological features of the different types of TGCC. Undifferentiated TGCC (seminomas, IGCN unclassified, and gonadoblastomas) are hypomethylated, whereas more differentiated TGCC (teratomas, yolk sac tumors, and choriocarcinomas) show a higher degree of methylation. Embryonal carcinomas show an intermediate pattern. Thus such parameters could be used to discriminate between seminoma and non-seminoma (Brait et al., 2012).

Such impact involved modifications of genes encoding for enzymes involved in DNA methylation. Consistent with cell type origin of TGCC, the Dnmts are mainly expressed in fetal testis and in the more undifferentiated cell type (spermatogonia) during adult normal spermatogenesis.

Regarding cancers, Dnmt1 was not expressed in seminoma, but upregulated in embryonal carcinoma (Omisanjo et al., 2007). In contrast, the expression of *Dnmt3a* was found up-regulated in TGCC compared to non-tumor testicular tissues (Yamada et al., 2004). The expression pattern of *Dnmt3b* has been deeply studied and show that it could be used as a predictive marker for relapse of stage I seminomas (Arai et al., 2012). Lastly, *Dnmt3l* was overexpressed in the non-seminoma tumors (Minami et al., 2010).

These changes in Dnmts expression will lead to main alteration of transcriptomic profile between tumors and normal tissues (Alagaratnam et al., 2011). Among them, two targets are of particular interest as they are genes of pluripotency. Indeed, early fetal germ cells and undifferentiated germ cell tumors have in common the expression of pluripotency markers such as the transcription factors *Nanog* and *Oct3/4*. Regarding *Nanog* promoter, it was found hypomethylated in spermatogonia and hypermethylated in sperm (Nettersheim et al., 2011). This selective repression might reflect that the cells need to suppress pluripotency in order to prevent malignant transformation. Finally, methylation of CpGs in the *Nanog* promoter in germ cell tumors and derived cell lines correlated to differentiation state. In the same line, the study *Oct3/4* showed that seminoma and embryonal carcinoma were hypomethylated (De Jong et al., 2007).

***Histone methylation***. Many enzymes are involved in histone methylation with specific or redundant level of methylation. These differences rely on the modified histone, targeted amino-acid residues and the number of methyl groups that are added to histone. These modifications are performed by several members of the HMT family. On those involved in spermatogenesis, the Suv39h1 and suv39h2 mediate histone H3 di and/or trimethylation at lysine 9 (Schotta et al., 2003). They are involved in meiosis as double KO mice present defects in male meiosis and highly pronounced apoptosis of stage IV spermatocytes during the transition from mid to late pachytene (O’Carroll et al., 2000; Peters et al., 2001).

G9a, a mammalian HMTase, is a candidate for H3-K9 methylation in non-heterochromatic loci (Tachibana et al., 2007). G9a is essential for early embryonic development and plays a dominant role in H3-K9 methylation of euchromatin. Its role was highlighted as mice lacking G9a are sterile, with germ cells undergoing apoptosis during the pachytene stage (Tachibana et al., 2007). Interestingly, it was demonstrated that G9a was a target of retinoid signaling pathway, a key regulator of germ cell differentiation, and that it was inhibited in the context of estrogenic exposure leading to the increased germ cell apoptosis induced by EDs (Volle et al., 2009).

Another HMT is the enhancer of zeste 2 (EZH2) which trimethylate histone H3 at the lysine 27 (Chang and Hung, 2012). It was demonstrate that during spermatogenesis, EZH2 is restricted to round spermatids in the perinuclear acrosome region (Lambrot et al., 2012). This localization is concomitant with the dramatic epigenetic reorganization that occurs during spermiogenesis leading to an extreme compaction of the chromatin.

DNA methylation, histone methylation are epigenetic modifications functioning in transcriptional control and have been implicated in the deregulation of gene expression in cancer. As mentioned above, the CIS cells present epigenetic profile similar to ES cells. Such properties of chromatin have been associated with a high transcriptional and proliferative activity. This is due to lower levels of DNA methylation and of histone methylation (H3K9me2 and H3K27me3; Almstrup et al., 2010). This is consistent with a lower expression of the gene encoding for the EZH2 in the TGCC compared to normal testis tissues (Hinz et al., 2010). This suggests that in TGCC EZH2 does not exert its often assumed oncogenic properties during malignant transformation and progression. High EZH2 levels in normal testicular tissue and the inverse association of its expression levels with the severity of spermatogenic failure point to its potential value as a molecular marker for spermatogenic defects and may indicate an important physiological role of EZH2 during intact spermatogenesis.

It has been suggested that histone H2A and H4 arginine 3 dimethylation might be a mechanism by which IGCNU and seminoma maintain the undifferentiated state; while loss of these histone modifications (Eckert et al., 2008) could be involved in the somatic differentiation observed in non-seminomatous tumors.

In healthy testis, the distribution of histone H3 methylation was dependent on the developmental stage of spermatogenic cells and in non-seminoma, histone H3-K4 and K9 methylation was detected in all histological subtypes (Lambrot and Kimmins, 2011). This suggested that histone H3-K4 and K9 methylation could be associated with abnormal gene expression in non-seminoma. Histone modifications determine epigenetic patterns of gene expression with methylation of histone H3 at lysine 4 (H3K4), and are often associated with active promoters. LSD1/KDM1 is a histone demethylase that suppresses gene expression by converting dimethylated H3K4 to mono- and unmethylated H3K4 (Wang et al., 2011). Interestingly, LSD1 protein level is highly elevated in pluripotent cancer cells and in human testicular seminoma tissues that express *Oct3/4* (Wang et al., 2011).

*Histone acetylation.* This is another histone modification that must also lead to gene repression. These modifications are performed by specific enzymes so-called histone deacetylase (HDCA). Such post-translation modifications are implicated in normal spermatogenesis (Fenic et al., 2008). Indeed, H3K9ac was shown in spermatogonia, spermatocytes, elongating spermatids, and ejaculated spermatozoa of fertile and infertile men (Steilmann et al., 2011). In spermatogonia, the stainings for H3K9ac, H3K18ac, and H3K23ac were strong. Then spermatocytes, the stainings for H3K9ac, H3K18ac, H3K23ac, and H3K4me3 were reduced in the preleptotene to pachytene stage, but in diplotene stage the stainings for H3K18ac, H3K23ac, and H3K4me3 seemed to become intense in later stages (Song et al., 2011a).

The main involvement of histone acetylation during spermatogenesis is the hyperacetylation of histone H4 during spermiogenesis (Dhar et al., 2012). This signal seems to play crucial role for removal of histones and their replacement by protamines, which is key feature for nucleus condensation, and thus formation of spermatozoa. There are multiple members classified in different subfamilies. Interestingly, choriocarcinomas showed generally high expression for all three class I HDAC isoforms (Gryder et al., 2012). However in contrast with other types of tumors, no diagnostic or prognostic values for HDAC1–3 in TGCC could be inferred (Fritzsche et al., 2011).

*Impact of TGCC treatment*. Some testis tumors are treated using cisplatin (Koychev et al., 2011). Recent report analyzed the impact of such treatment on the integrity of spermatozoa chromatin in rats (Maselli et al., 2012). As expected, the cisplatin treatment lead to susceptibility of DNA to denaturation and the number of strand breaks were significantly increased in mature sperm. After a recovery period, it was noted that mature sperm did not show significant DNA damages. However, the protamination level of the sperm of these animals was significantly decreased. This was associated with an up-regulation of the histones H1.2, H4, H2A1, and H2B1A. This suggests long-term effect of cisplatin treatment that could have consequences for progenies even after the arrest of cancer cure.

***Small non-coding RNA***. Next to these well studied epigenetic processes, it also appears that microRNA and small RNA play important roles in both germ cell differentiation and transmission to subsequent generations (He et al., 2009; Suh and Blelloch, 2011; Buckley et al., 2012). Indeed, achieving the correct spatial and temporal expression of germ cell-specific genes is fundamental to the production of spermatozoa (Song et al., 2011b). Notably, for the regulation of genes involved in the repression of protein translation is central to many embryonic processes, and is particularly active during spermatogenesis.

The miRNA and siRNA are generated by the nuclear RNase III enzyme Drosha and the cytoplasmic RNase III enzyme Dicer (Papaioannou and Nef, 2010). The involvement of these particular RNA has been highlighted by the generation of mouse models invalidated for genes encoding Drosha or Dicer (Korhonen et al., 2011; Wu et al., 2012). These invalidations led to sterility due to disrupted spermatogenesis characterized by depletion of spermatocytes and spermatids leading to oligoteratozoospermia or azoospermia. miRNAs mostly act by destabilizing target mRNAs or inhibiting their translation. Next to this, the PIWI-interacting RNAs (piRNAs) are predominantly expressed in the germ cell lineage (Kibanov et al., 2012). The analyses on this particular class of RNA suggest that they have a potential role in epigenetic regulation of cell polarization. Moreover piRNA seem to be involved in silencing of transposon expression.

Many miRNA and siRNA have been implicated in the different steps of spermatogenesis. Among them, miR-449 and miR-34 seems to have common targets on the E2F signaling pathway which is mainly involved in the regulation of male germ cell development (Bao et al., 2012). Mir-17-92 (Mirc1) cluster and Mir-106b-25 (Mirc3) cluster miRNAs were suggested to cooperate in regulating spermatogonial development (Tong et al., 2012). The overexpression of miR-184 was demonstrated to promote the proliferation of a germ cell line, GC-1spg (Wu et al., 2011). It was also shown that transient inhibition of miR-21 in SSC-enriched germ cell cultures increased the number of germ cells undergoing apoptosis (Niu et al., 2011).

Interestingly, several miRNAs are unique to testis. Regarding the involvement of miR199a-5p was highlighted by the study showing an inverse relationship between miR-199a-5p and embryonal carcinoma antigen podocalyxin-like protein 1 (PODXL) expression (Cheung et al., 2011). This suggests that PODXL must be a downstream effector mediating the action of miR199a-5p. This is of particular interest as PODXL, an anti-adhesive protein, is expressed in aggressive tumors.

Next to this, miR-371-373 and miR-302 clusters are overexpressed in malignant TGCC (Novotny et al., 2012). It downregulates mRNAs involved in biologically significant pathways involved in cellular senescence induced by oncogenic stress.

Among other examples, miRNA-383 expression is downregulated in the testes of infertile men with MA (Lian et al., 2009). These results suggest that it functions as a negative regulator of proliferation, in part, through inactivation of the pRb pathway. Thus an abnormal expression of miRNA-383 may potentiate the connections between male infertility and testicular germ cell tumor.

*Therapy issues*. A percentage of tumors are resistant to cisplatin treatment. It seems to be associated with the high cytoplasmic expression of p21. Interestingly, it was demonstrated that there is an inverse association between cisplatin resistance and the expression of Oct4 and miR-106b (Koster et al., 2010). Thus, it was suggested that modulation of the Oct4/miR-106b/p21 pathway could open new perspectives in the treatment of chemoresistant TC.

## CONCLUSIONS/PERSPECTIVES

The increasing incidence of reproductive tract diseases and particularly TC, during the last decades, is of concern. Indeed, even if it is a well curable disease with a good 5-year survival rate, it affects men during the time of their reproductive life (between 15 and 40), suggesting that it may affect both fertility and also health of the progeny. It cannot be exclude that germ cells generated at the beginning of carcinogenesis could transmit altered DNA material, due to genetic, epigenetic perturbation. This higher rate of appearance for TC cases is supposed to be associated with exposure to environmental chemicals. This also need to be deeply studied as in our modern society, men are exposed to an increasing amount of chemicals. This suggests that the incidence of TC could be even more important in the future decades. All these potential consequences point out the importance to study the involved mechanisms in appearance, and progression of TGCC. This means that we have to better understand the etiology for such cancers.

In long-term perspective, an increased knowledge of genetic, epigenetic, and gene expression patterns correlated with data of anatomopathology will lead to a better definition and understanding of the pathology. It also suggests that there is a need to analyze patients in a case by case approach in order to identify genetic, epigenetic alterations, and modifications of gene expression patterns. This will help to propose personalized therapy that would probably help in improving survival rate and life quality of survivors and avoid relapse.

## Conflict of Interest Statement

The authors declare that the research was conducted in the absence of any commercial or financial relationships that could be construed as a potential conflict of interest.
